# Monitoring Fruit Growth and Development in Apricot (*Prunus armeniaca* L.) through Gene Expression Analysis

**DOI:** 10.3390/ijms25169081

**Published:** 2024-08-21

**Authors:** Germán Ortuño-Hernández, María Sánchez, David Ruiz, Pedro Martínez-Gómez, Juan Alfonso Salazar

**Affiliations:** Department of Plant Breeding, Centro de Edafología y Biología Aplicada del Segura—Consejo Superior de Inbvestigaciones Científicas (CEBAS-CSIC), Campus Universitario Espinardo, E-30100 Murcia, Spain; gortuno@cebas.csic.es (G.O.-H.); espejomaria695@gmail.com (M.S.); druiz@cebas.csic.es (D.R.); jasalazar@cebas.csic.es (J.A.S.)

**Keywords:** apricot, fruit development, breeding, gene expression, nutraceutical properties, RT-qPCR

## Abstract

The main objective of this study was to monitor apricot development and ripening through gene expression analysis of key candidate genes using the RT-qPCR technique. Eight apricot cultivars were selected to analyze phenological and genetic patterns from pre-ripening stages through to postharvest. In addition, 19 selected genes were analyzed in the contrasting cultivars ‘Cebas Red’ and ‘Rojo Pasión’ in different stages (two preharvest stages S1 and S2, one harvest stage S3, and two postharvest stages S4 and S5). This pool of genes included genes related to fruit growth and ripening, genes associated with fruit color, and genes linked to the fruit’s nutraceutical aspects. Among the studied genes, *Polygalacturonase* (*PG*), *Pectin methylesterase* (*PME*), *Aminocyclopropane-1-carboxylate synthase* (*ACS*), and *Myo-inositol-1-phosphate synthase* (*INO1*) were directly related to fruit maturation and quality. Significant differential expression was observed between the cultivars, which correlated with variations in firmness, shelf life, and sensory characteristics of the apricots. ‘Rojo Pasión’ displayed high levels of *PG*, associated with rapid maturation and shorter postharvest shelf life, whereas ‘Cebas Red’ exhibited lower levels of this gene, resulting in greater firmness and extended shelf life. Genes *CCD4*, *CRTZ*, and *ZDS*, related to carotenoids, showed varied expression patterns during growth and postharvest stages, with higher levels in ‘Rojo Pasión’. On the other hand, *Sucrose synthase* (*SUSY*) and *Lipoxygenase* (*LOX2*) were prominent during the postharvest and growth stages, respectively. Additionally, *GDP-L-galactose phosphorylase* (*VTC2_5*) was linked to better postharvest performance. This research provides valuable insights for future breeding initiatives aimed at enhancing the quality and sustainability of apricot cultivation.

## 1. Introduction

Apricot (*Prunus armeniaca* L.) is a stone fruit belonging to the *Prunus* genus, which produces an edible mesocarp of great economic and nutritional significance in various regions worldwide. This commercially valued fruit is distinguished by its exquisite, sweet flavor—typically measuring around 10–15º Brix [1]—its attractive color, and its abundance of nutrients, notably its antioxidant properties and phenolic compound content [2]. The apricot fruit displays a remarkable diversity in quality traits due to its extensive genetic diversity [3]. This diversity streamlines the efforts of breeding programs, which aim to explore agronomic aspects, fruit quality, postharvest characteristics, and resistance to both biotic and abiotic stresses. Ultimately, the overarching objective is to develop novel cultivars that can mitigate production costs, enhance crop yields, and elevate the overall quality of the final product [4]. Currently, apricot cultivation spans 65 countries worldwide, with Turkey emerging as the leading producer (713 thousand tons), followed by Uzbekistan (487 thousand tons), Iran (315 thousand tons), Italy (230 thousand tons), France (214 thousand tons), and Spain (131 thousand tons), thanks to the ideal soil and climatic conditions for its growth [5]. Consequently, its cultivation is predominantly spread from Central Asia to Europe, and the increasing global market demand has driven the need for a deeper understanding of its development and ripening to enhance desirable fruit characteristics.

On the other hand, analysis of the development and ripening process is a key objective of plant breeding programs. During this period, the fruit undergoes important physiological and biochemical changes that significantly influence its quality. Thus, a thorough understanding of the molecular mechanisms associated with fruit growth and ripening enables the development of strategies to produce improved cultivars with enhanced fruit quality and shelf life [6,7]. The fruit development process is a complex phenomenon that spans from flowering to full ripening. During this period, the fruit undergoes a series of morphological, biochemical, and physiological changes that dictate its quality, flavor, texture, and commercial value [8]. In the case of stone fruit trees within the Rosaceae family, such as peach and apricot trees, a double sigmoid growth pattern is observed [9]. This type of fruit growth is characterized by two phases of rapid growth (S1 and S3) separated by a phase of slow growth (S2). Transcriptomic analysis studies have identified genes related to cell development that play a pivotal role in determining the fruit growth pattern [10]. Fruit ripening is a highly coordinated process that coincides with seed maturation, encompassing the final stages of growth and development until the onset of senescence [11]. During this stage, softening and/or lignification of the pericarp layers occur, alongside the accumulation of sugars, acids, pigments, and the release of volatile compounds, enabling the fruit to attain its characteristic quality attributes, making it appealing for human consumption [12,13]. Genes expressed during the ripening process are associated with firmness loss [14], aroma [15], flavor [16], ethylene [17], carotenoids [18], flavonoids [19], and anthocyanins analysis [20]. All these changes result from a coordinated modulation of the gene expression network regulated by complex and interconnected mechanisms affected by internal and external factors [21]. Advances in molecular biology have opened new perspectives for understanding the underlying mechanisms of fruit development and ripening. The study of gene expression, in particular, has emerged as a powerful tool for unraveling the molecular processes that regulate fruit biology [22]. By identifying and characterizing genes involved in apricot development and ripening, researchers can obtain valuable insights into metabolic pathways, regulatory networks, and environmental factors that influence crop quality and productivity [23]. In this context, monitoring the expression of the major genes involved in the key active pathways of the ripening process and their phenotypic correlation can elucidate the key genes responsible for fruit quality traits in apricot [24].

The main objective of this study was to monitor apricot development and ripening by analyzing gene expression of key candidate genes using the RT-qPCR technique. Our approach combines traditional phenology with molecular tools to comprehensively understand the events occurring from the pre-ripening stages to the postharvest phases. Through this integrated approach, we aim to contribute to deepening the knowledge of apricot biology and provide valuable information on its cultivation, management, and improvement in the context of climate change and increasing demand.

## 2. Results

### 2.1. Pomological Monitoring of Fruit Development and Ripening

**Figure 1:**
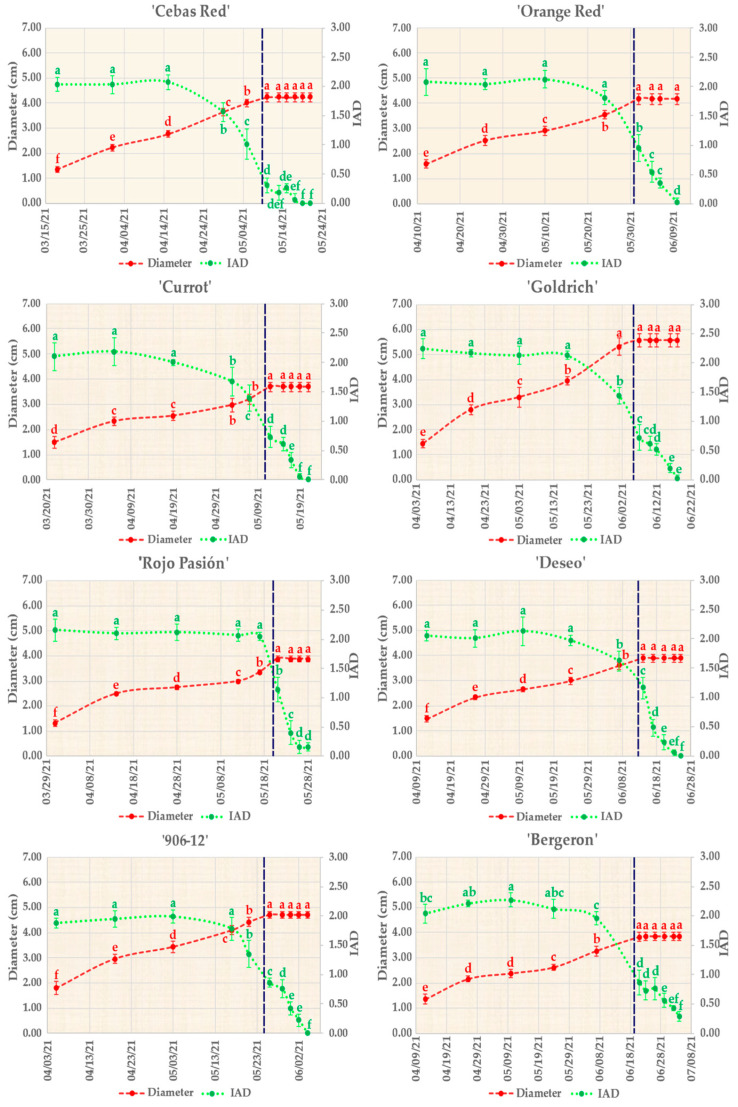
Fruit growth (diameter in red line) and chlorophyll index (I_AD_, green line) during the fruit development period in the different assayed apricot cultivars. Different letters indicate significant differences between the days for each genotype according to the Tukey test (*p*-value < 0.05).

In the analysis of the growth and development of the fruit, the diameter and chlorophyll index (I_AD_) were assessed at stages prior to physiological ripening with the aim of observing the behavior of each apricot cultivar (Figure 1).

Regarding fruit growth, the growth curve of all cultivars resembles the characteristic double sigmoid pattern of stone fruits. Notably, the ‘Rojo Pasión’ cultivar best exhibits this behavior, while ‘Cebas Red’ shows a less pronounced trend. This parameter thus reflects some variations in the fruit growth trends among cultivars. It is worth mentioning that ‘Goldrich’ was the cultivar with the largest size (approximately 5.5 cm in diameter), whereas ‘Currot’ had a diameter of <4 cm, making it the smallest harvested fruit. The other cultivars exhibited similar diameters, ranging between 4 and 5 cm.

As for the chlorophyll evolution through the I_AD_ index, in the early stages of fruit development, it remained relatively stable with an approximate value of 2 in all cultivars. Subsequently, a rapid decline occurred, in which three groups can be differentiated according to the I_AD_ value at the time of commercial ripeness: ‘Rojo Pasion’ and ‘Deseo’ with I_AD_ > 1, ‘Currot’, ‘906-12’, ‘Orange Red’, ‘Goldrich’, and ‘Bergeron’ with I_AD_ between 0.5 and 1, and finally ‘Cebas Red’ with I_AD_ < 0.5. This commercial ripeness was considered when the fruit color reached at least three-quarters of the fruit’s surface and the fruit developed its eating ripeness after early harvest. Therefore, we can assign an I_AD_ value for commercial harvesting to each cultivar.

**Figure 2:**
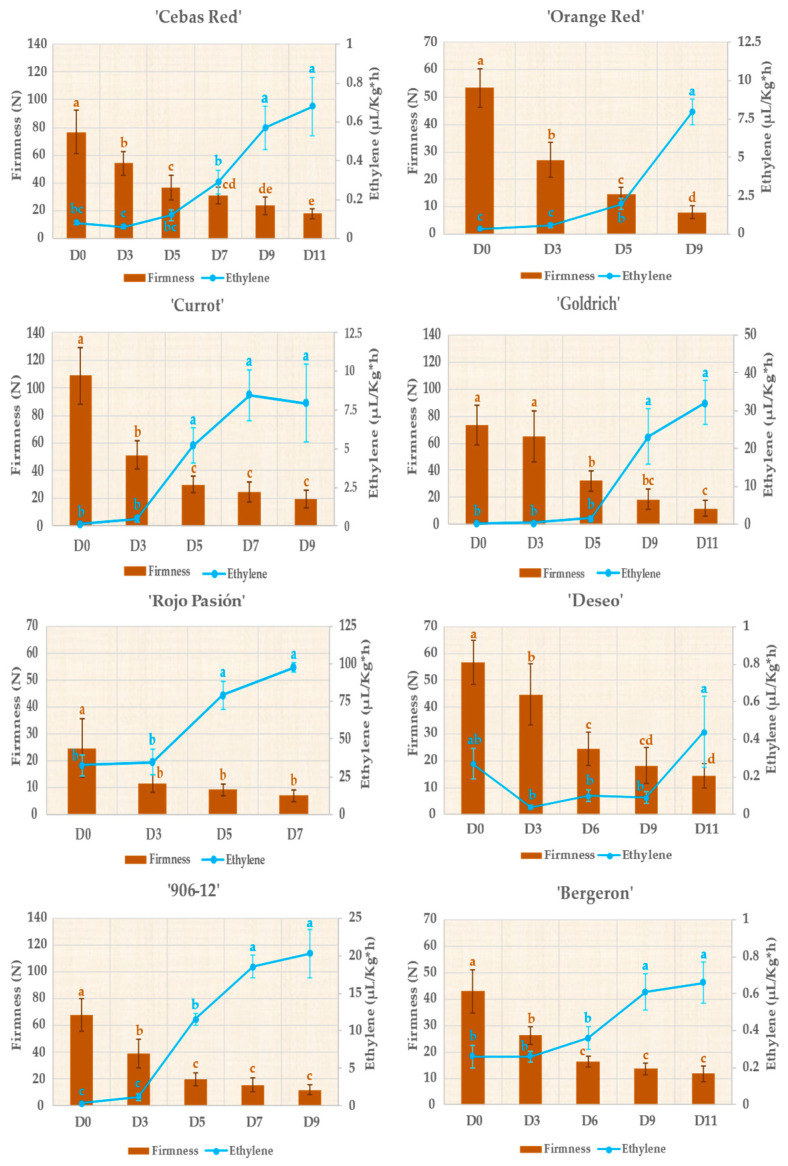
Firmness (brown bars) and ethylene production (blue line) during the postharvest period of the assayed apricot cultivars. Different letters indicate significant differences between the days for each genotype according to the Tukey test (*p*-value < 0.05).

On the other side, these results suggest a dynamic of growth in diameter and changes in the I_AD_ that differ among apricot cultivars, which could reflect genetic and adaptive differences in fruit development. Other characteristics evaluated to study the postharvest behavior of the fruit included firmness and ethylene production (Figure 2). Regarding ethylene production, all cultivars reached their peak values in the final days postharvest. However, ethylene production varies among the different cultivars, which significantly determines differences in fruit shelf life. The cultivars that emit the least amount of ethylene are ‘Cebas Red’, ‘Deseo’, and ‘Bergeron’, with maximum values of 0.7, 0.4, and 0.7 µL/kg × h, respectively, coinciding with a lower fruit softening rate, as previously mentioned. Conversely, ‘Rojo Pasión’ is the cultivar that produced the highest amount of ethylene, with a peak value of 97.5 µL/kg × h, coinciding with a much shorter shelf life.

### 2.2. Monitoring Fruit Quality Traits during Postharvest

Pomological traits evaluated at harvest revealed considerable variability among the 8 cultivars studied (Figure 2 and Table 1). In terms of fruit weight, ‘Goldrich’ was the only cultivar whose weight exceeded 100 g, while ‘Currot’ did not reach 40 g. The rest of the cultivars had similar values, indicating a clear correlation between fruit diameter and weight. Regarding fruit color at harvest, ‘Cebas Red’ had the most orange skin and flesh color (h° ≈ 74) among the cultivars, unlike ‘Currot’, which was characterized by its yellowish skin and flesh (h° ≈ 100). Fruit firmness at harvest varied widely since fruits were harvested based on the color criterion. Although most showed a firmness value between 50 and 80 N, the most contrasting cultivars were ‘Currot’ (109 N) and ‘Rojo Pasión’ (24 N). As for the percentage of red blush, ‘Cebas Red’, ‘Orange Red’, and ‘906-12’ reached values of around 25%. On the other hand, the most intense red values were achieved in these same cultivars along with ‘Deseo’ (Table 1). The soluble solids content at harvest time ranged from 8 to 13 °Brix, with ‘Cebas Red’ having the lowest sugar content, while the ‘Deseo’ cultivar reached values of almost 13 °Brix. However, it should be noted that, in general, sugar levels were quite low due to a very early harvest time for commercial purposes. Acidity at the time of harvest ranged between 1 and 3 g of malic acid/100 mL, with ‘Goldrich’ being by far the most acidic cultivar (2.65 g/100 mL), while ‘Orange Red’, ‘Cebas Red’, and ‘Rojo Pasion’, with values around 1 g/100 mL, were the least acidic, favoring a better balance between sugars and acidity, as these latter cultivars showed very low levels of soluble solids. 

On the other hand, if we consider the evolution of the fruit color (Supplementary Figure S1), all cultivars experienced at least a decrease in the h° values, especially in the skin, with ‘Currot’ (from yellow to slightly orange tones) and the selection ‘906-12’ (from slightly orange to intense orange) showing the highest decline in h° values. However, no differences were found when we paid attention to the evolution of soluble solids (Supplementary Figure S2) from harvest time to the last postharvest day. In terms of acidity (Supplementary Figure S2), these differences were elevated, especially in ‘906-12’, ‘Cebas Red’, ‘Currot’, ‘Goldrich’, and ‘Orange Red’, which showed a significant decrease between harvest time and the end of postharvest.

**Figure 3:**
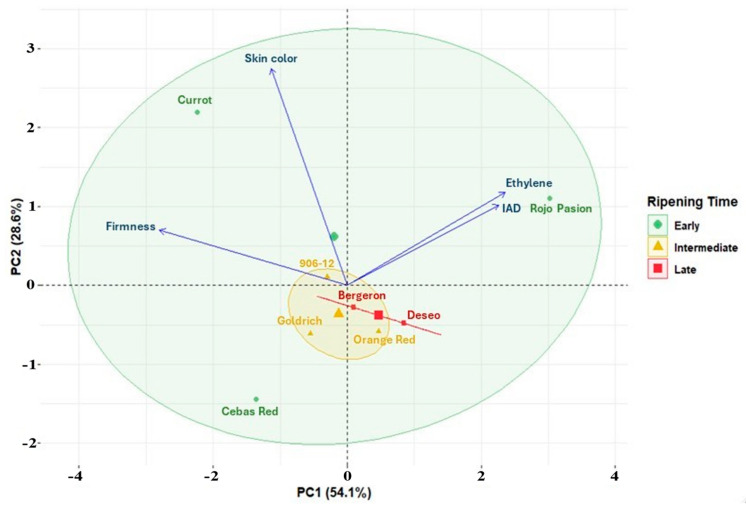
Principal component analysis (PCA) of firmness, ethylene production, I_AD_, and skin color traits in the assayed apricot cultivars regarding ripening time, early, intermediate and late.

To better understand the differences between the eight studied apricot cultivars, we conducted a principal component analysis (PCA), depicted in Figure 3. This analysis highlights the variations between these cultivars in terms of firmness, ethylene production, I_AD_, and skin color. The majority of the variation is explained by the *x*-axis (PC1), with ethylene production, I_AD_, and firmness being the most determinant traits. Conversely, the skin color variable contributes more significantly to the construction of the y component (PC2).

Based on the observations from the PCA in Figure 3 and of previous results, ‘Rojo Pasión’ stands out among the studied cultivars for its higher ethylene production, reaching levels of 97.5 µL/kg × h at the end of the postharvest period. However, ‘Cebas Red’ exhibits one of the lowest ethylene production values (0.7 µL/kg × h) (Figure 2). Conversely, ‘Cebas Red’, along with ‘Currot’, is notable for maintaining higher firmness, with a value of 17.91 N on the last postharvest day, whereas ‘Rojo Pasión’ had the lowest firmness levels at the end of the postharvest period (7.06 N) (Figure 2). Moreover, based on the I_AD_ value at the time of commercial ripeness, ‘Rojo Pasión’ and ‘Deseo’ had I_AD_ values above one, while ‘Cebas Red’ had a value below 0.5. Therefore, ‘Cebas Red’ and ‘Rojo Pasión’ are the most contrasting cultivars, and were selected for a differential gene expression study during the growth, ripening, and postharvest stages of the fruit. This study aims to determine the molecular-level differences between these two cultivars and to enhance our understanding of the factors associated with fruit growth and ripening.

### 2.3. Gene Expression Analysis in Relation to Fruit Development and Ripening

**Figure 4:**
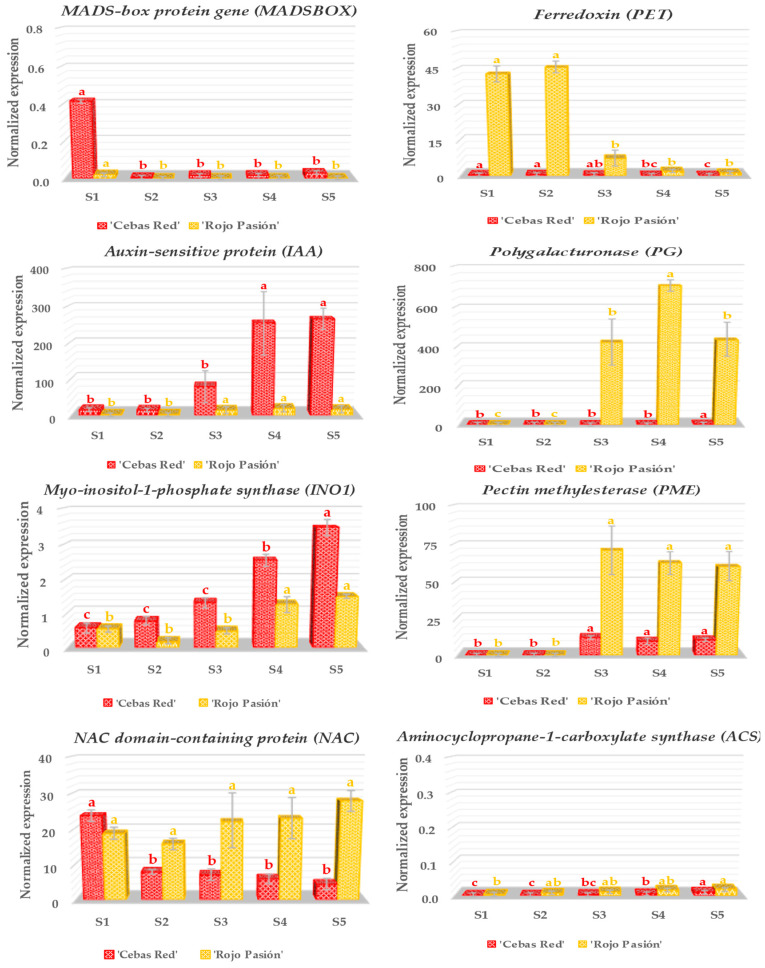
Gene expression analysis in relation to fruit growth and ripening in ‘Cebas Red’ and ‘Rojo Pasión’ cultivars at different growth stages: two pre-harvest stages (S1 and S2), harvest stage (S3), and two postharvest stages (S4 and S5). Vertical lines indicated standard deviations. Different letters indicate significant differences between stages for the genotypes according to the Tukey test (*p*-value < 0.05).

**Figure 5:**
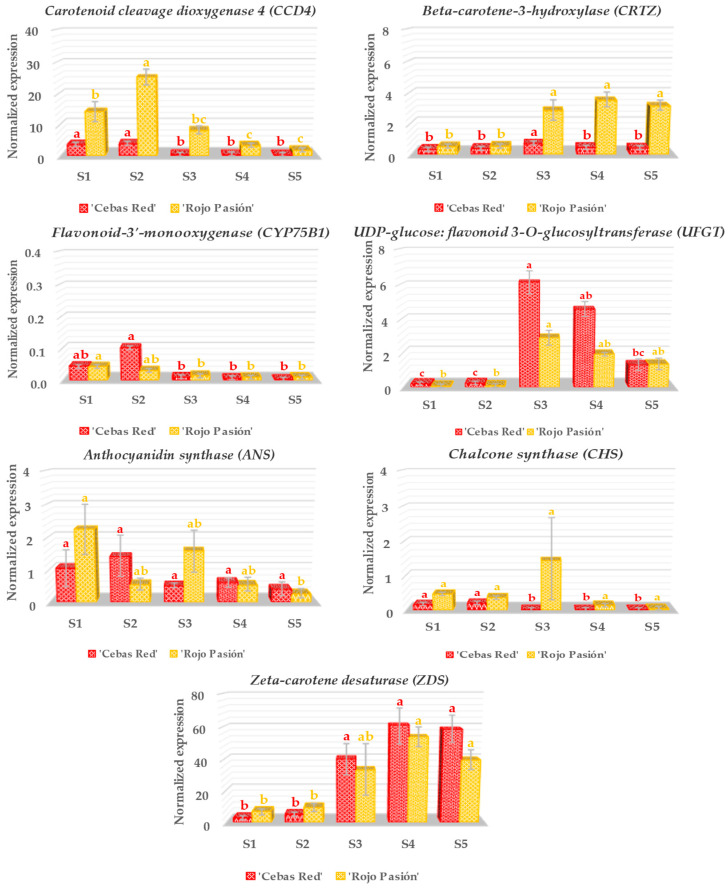
Gene expression analysis in relation to fruit color in ‘Cebas Red’ and ‘Rojo Pasión’ cultivars at different growth stages: two pre-harvest stages (S1 and S2), harvest stage (S3), and two postharvest stages (S4 and S5). Vertical lines indicated standard deviations. Different letters indicate significant differences between stages for the genotypes according to the Tukey test (*p*-value < 0.05).

**Figure 6:**
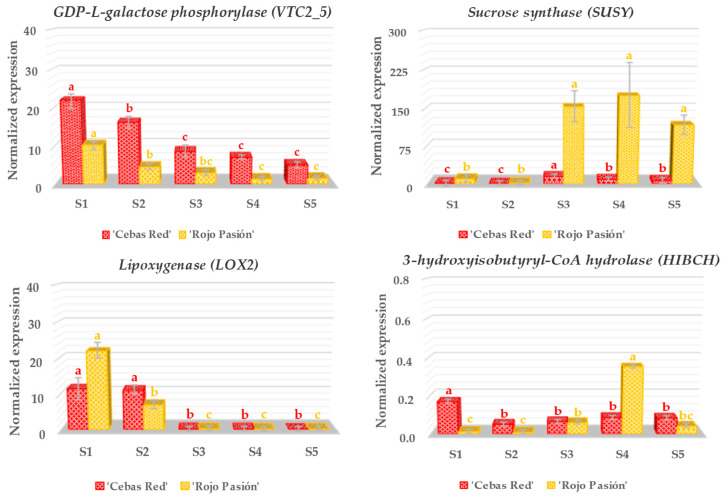
Gene expression analysis in relation to the nutraceutical properties of the fruit in ‘Cebas Red’ and ‘Rojo Pasion’ cultivars at different growth stages: two pre-harvest stages (S1 and S2), harvest stage (S3), and two postharvest stages (S4 and S5). Vertical lines indicated standard deviations. Different letters indicate significant differences between stages for the genotypes according to the Tukey test (*p*-value < 0.05).

Nineteen genes related to fruit development and ripening, including *Auxin-sensitive protein* (*IAA*), *Ferredoxin* (*PET*), *Myo-inositol-1-phosphate synthase* (*INO1*), *MADS-box protein gene* (*MADSBOX*), *NAC domain-containing protein* (*NAC*), *Polygalacturonase* (*PG*), *Pectin methylesterase* (*PME*), *Aminocyclopropane-1-carboxylate synthase* (*ACS*), *Anthocyanidin synthase* (*ANS*), *UDP-glucose: flavonoid 3-O-glucosyltransferase* (*UFGT*), *Beta-carotene-3-hydroxylase* (*CRTZ*), *Carotenoid cleavage dioxygenase 4* (*CCD4*), *Zeta-carotene desaturase* (*ZDS*), *Chalcone synthase* (*CHS*), *Flavonoid-3’-monooxygenase* (*CYP75B1*), *3-hydroxyisobutyryl-CoA hydrolase* (*HIBCH*), *GDP-L-galactose phosphorylase* (*VTC2_5*), *Sucrose synthase* (*SUSY*), and *Lipoxygenase* (*LOX2*) were analyzed in the contrasting cultivars ‘Cebas Red’ and ‘Rojo Pasión’ (Figure 4, Figure 5 and Figure 6). 

Based on gene function (Supplementary Table S1), they were categorized into three groups: genes related to fruit growth and ripening (*IAA*, *PET*, *INO1*, *MADSBOX*, *NAC*, *PG*, *PME*, and *ACS*), genes related to fruit color (*ANS*, *UFGT*, *CRTZ*, *CCD4*, *ZDS*, *CHS*, and *CYP75B1*), and genes related to the nutraceutical aspect of the fruit (*HIBCH*, *VTC2_5*, *SUSY*, and *LOX2*). Additionally, of the five stages considered for analysis, two are pre-harvest growth stages (S1 and S2), another corresponds to commercial ripeness determined by characteristic color criterion (S3), and the final two stages are postharvest (S4 and S5).

Regarding the genes predominantly affecting fruit growth and ripening (Figure 4), the *Ferredoxin* (*PET*) gene exhibited high normalized relative expression (NRE) in the early stages, S1 and S2, in ‘Rojo Pasión’ compared to ‘Cebas Red’. This gene was the only one from this first group analyzed that acted markedly during the fruit growth stage. When focusing on commercial maturity (S3) and the postharvest stages (S4 and S5), the genes *Auxin-sensitive protein* (*IAA*) and *Myo-inositol-1-phosphate synthase* (*INO1*) showed differential expression between the cultivars, with ‘Cebas Red’ displaying higher expression levels compared to the other cultivar. Conversely, *Polygalacturonase* (*PG*) and *Pectin methyl esterase* (*PME*) displayed higher expression levels in ‘Rojo Pasión’. Surprisingly, the *NAC domain-containing protein* (*NAC*) displayed different trends depending on the cultivar. ‘Cebas Red’ showed decreasing expression levels from S1 to S5, with NRE from 24 to 4; meanwhile, ‘Rojo Pasión’ exhibited a slight upward trend from S1 to S5, with levels from 18 to 28. Finally, the *MADS-box protein gene* (*MADSBOX*) and *Aminocyclopropane-1-carboxylate synthase* (*ACS*) showed minimal expression signals across the different stages.

Regarding the genes related to color (Figure 5), three classes can be distinguished based on the natural pigment they affect: carotenoids, flavonoids, or anthocyanins. *Carotenoid cleavage dioxygenase 4* (*CCD4*), *Beta-carotene-3-hydroxylase* (*CRTZ*), and *Zeta-carotene desaturase* (*ZDS*) are three genes associated with the metabolic pathways for carotenoids. The *CCD4* gene was expressed during the fruit growth stage, exhibiting a higher level at S2 than at S1 in ‘Rojo Pasión’ in contrast to ‘Cebas Red’, which followed the same pattern but with much lower levels. On the other hand, despite the low expression levels, the *CRTZ* gene exhibited higher expression during the postharvest stages, especially in ‘Rojo Pasión’. Lastly, the *ZDS* gene, displaying a trend similar to *CRTZ*, had the highest expression levels, with similar NRE expression in both ‘Cebas Red’ and ‘Rojo Pasión’. In terms of flavonoids and anthocyanins, the genes *Chalcone synthase* (*CHS*), *Flavonoid-3’-monooxygenase* (*CYP75B1*), and *Anthocyanidin synthase* (*ANS*) showed very low NRE values across the five stages under study. However, *UDP-glucose: flavonoid 3-O-glucosyltransferase* (*UFGT*) experienced a significant increase from S2 to S3, followed by a decrease in the postharvest stage (from S3 to S5), whereas ‘Cebas Red’ demonstrated slightly higher NRE values than ‘Rojo Pasión’.

Regarding the studied genes related to the nutraceutical properties of apricots (Figure 6), *Sucrose synthase* (*SUSY*) stands out, with significantly higher expression levels in ‘Rojo Pasión’ compared to ‘Cebas Red’, especially from the S3 to S5 stages. Meanwhile, the *GDP-L-galactose phosphorylase* (*VTC2_5*) gene exhibited a decreasing trend from S1 to S5, with ‘Cebas Red’ exhibiting higher expression levels. Finally, *3-hydroxyisobutyryl-CoA hydrolase* (*HIBCH*) and *Lipoxygenase* (*LOX2*), two genes mainly linked to fruit’s aroma, were analyzed. However, significant differences between cultivars were observed only for the *LOX2* gene during stages S1 and S2 of growth, remaining constant at 10 units of NRE for ‘Cebas Red’, while ‘Rojo Pasión’ decreased from 22 to 7.

## 3. Discussion

### 3.1. Monitoring Fruit Development, Ripening, and Shelf Life

In terms of fruit growth, all cultivars followed the characteristic double sigmoid pattern typical of stone fruits [9]. However, little is known about the differences among cultivars that vary in their fruit development periods. In our case, ‘Cebas Red’ is an early cultivar characterized by a very short growth cycle. For this reason, the double sigmoid trend disappears, resulting in faster growth and a reduced stone hardening stage, leading to lower seed development. This should be considered in breeding programs, as the success of crosses using cultivars with shorter fruit development periods as the female parent could result in a lower number of viable seedlings, as happens in other fruit trees such as pears [25].

Regarding chlorophyll evolution based on the I_AD_ index, it remained relatively stable at approximately two across all cultivars in the early stages of fruit development. Subsequently, a rapid decline occurred, coinciding with a change in skin color. However, chlorophyll content and fruit skin color are genotype-dependent, as apricot cultivars vary in skin color from light yellow to intense orange or even red, depending on the cultivar, although red blush coverage is influenced by solar incidence. A peculiar example is ‘Cebas Red’, which was harvested with the lowest I_AD_ of around 0.3, being the cultivar with the most intense orange skin. Conversely, ‘Rojo Pasión’ had an I_AD_ above 1 and exhibited a less intense orange skin color. Thus, if we compare fruit firmness and I_AD_, ‘Cebas Red’ was collected at 70 N whereas ‘Rojo Pasión’ was around 25 N. This demonstrates that although lower values of firmness usually correspond with lower levels of chlorophyll and, therefore, a lower I_AD_ index, each cultivar behaves differently. Thus, the I_AD_ index is not necessarily associated with a specific firmness when comparing different cultivars. Therefore, to establish an I_AD_ as a harvest criterion based on chlorophyll content, it is necessary to do so independently for each cultivar [26]. 

The PCA reveals significant variations regarding firmness, ethylene production, Index of Absorbance Difference (I_AD_), and skin color. On the other hand, during the postharvest period, there is a marked loss of firmness, leading to what is known as the softening rate. Additionally, climacteric fruits such as apricots exhibit an increase in ethylene biosynthesis during their ripening process [27]. In our assay, despite the cultivars being harvested with different firmness levels due to using color criteria for commercial purposes, the softening rate, and therefore the shelf life, of each cultivar varied. ‘Currot’ was harvested at around 100 N and ‘Cebas Red’ at around 70 N, with ‘Cebas Red’ achieving a more extended shelf life (11 days) compared to ‘Currot’ (9 days). These results demonstrate that ‘Currot’ displayed a higher softening rate than ‘Cebas Red’ despite starting with almost 30 N more. Another case is ‘Rojo Pasión’, which had the shortest shelf life. This result can be explained by the very low initial firmness of ‘Rojo Pasión’ (25 N). However, if we tried to harvest this fruit with higher firmness, it would be completely green and probably would never change to its consumption color. For this reason, we can never say that the harvest criterion for a cultivar is strictly based on firmness, I_AD_, or color; instead, each cultivar has optimal values depending on whether we seek consumption or commercial maturity. In terms of ethylene production, all cultivars reached peak values in the final days of postharvest. Overall, cultivars with the lowest ethylene production, such as ‘Cebas Red’, correspond to the longest shelf-lives, in contrast to ‘Rojo Pasión’, which had by far the highest ethylene production [28].

On the other hand, in terms of the evolution of fruit quality traits, the fruit color of all cultivars experienced, to a greater or lesser extent, a decrease in h° values, with ‘Currot’ and ‘906-12’ showing the highest decline. However, no significant or minimal differences were found in terms of soluble solids content and acidity. This should be considered to balance the goal of obtaining better postharvest performance by harvesting before full maturity and achieving maximum sensory attributes. In this study, we found that important traits such as soluble solids and acidity hardly evolve if the fruit is harvested very early.

### 3.2. Analysis of Genes Linked to Fruit Growth and Ripening

The observed differential expression in genes associated with fruit growth and ripening, such as *PG*, *PME*, *IAA*, and *PET*, confirms and expands upon scientific findings [22,29,30,31]. *PG* and *PME* genes played a crucial role in the ripening and softening of fruits in many plant species, including apricot [32]. Both genes are involved in pectin modification, a major component of fruit cell walls, but act differently during this process. *PME* activity is considered an important preliminary step that renders pectin more susceptible to degradation by *PG* and other pectinases, thereby directly affecting fruit texture and postharvest quality [14]. In our assay, in agreement with the observed results at phenological and RNA-seq levels [31], ‘Rojo Pasión’ had the highest NRE, in contrast to ‘Cebas Red’, especially at the S3, S4, and S5 stages. Additionally, the differential expression of PG and PME in selected apricot cultivars correlates with firmness values from the harvest to postharvest stages analyzed (Figure 2), corresponding to the lowest firmness values for ‘Rojo Pasión’, with the highest NRE for both genes. Consequently, the expression patterns of these genes could provide useful indicators for selecting fruits with improved shelf life and postharvest quality.

The *Auxin-sensitive protein* (*IAA*) gene is part of a gene family that plays a crucial role in the response and signaling of auxin, a plant hormone essential for plant growth and development [33]. Auxin influences processes such as cell elongation, tissue differentiation, cell division, and responses to environmental stimuli [34]. The *IAA* genes, due to their sensitivity to auxin, are important mediators in these processes, acting rapidly to regulate gene expression in response to changes in auxin levels. In the context of apricot, as the fruit ripens, the decrease in auxin is one of the hormonal changes that signal the transition from active growth to the ripening phase. Modulating the expression of *IAA* during apricot ripening could therefore influence these processes by adjusting the plant’s response to auxin and mediating the coordination of ripening. In the current study, the results suggest that this gene could be an important component in the hormonal regulation network controlling fruit ripening among different cultivars since significant differences were found between ‘Rojo Pasión’ and ‘Cebas Red’. Thus, ‘Cebas Red’ showed higher NRE values than ‘Rojo Pasión’, especially after harvest, which is undoubtedly linked to better postharvest performance. Furthermore, the expression patterns of *IAA* could reflect specific adaptations of each cultivar to its environment and ripening signals, which have significant implications for apricot cultivation and postharvest.

The *Ferredoxin* (*PET*) gene, involved in photosynthetic and electron transfer processes, plays a significant role in the plant’s energy metabolism. While *PET*’s primary focus is on its involvement in photosynthesis, its implication in fruit development and ripening, such as in apricot, may be indirect through energy metabolism and stress response. During fruit growth, a significant amount of energy is required for the synthesis of organic compounds, including those contributing to apricot flavor, color, and nutritional value. By influencing photosynthesis efficiency, *PET* indirectly affects the availability of energy for these processes. Moreover, by participating in photosynthetic energy production, it can also influence the mitigation of oxidative stress through the regulation of reactive oxygen species production [35]. In this assay, the *PET* gene showed especially higher expression in ‘Rojo Pasión’ during fruit growth, while ‘Cebas Red’ exhibited very low NRE values. This could mean that the higher NRE of the PET gene is linked to rapid fruit softening in ‘Rojo Pasión’, and its low expression is associated with good postharvest performance in ‘Cebas Red’. 

The *Myo-inositol-1-phosphate synthase* (*INO1*) gene plays a crucial role in Myo-inositol metabolism in apricots, essential for cellular processes such as fruit growth and development, cell signaling, and response to osmotic stress [36,37]. *INO1* catalyzes the conversion of glucose-6-phosphate into myo-inositol-1-phosphate, the first step in Myo-inositol synthesis, a precursor to important compounds like membrane phospholipids, phytates, and secondary messengers [38]. This synthesis is vital for cell growth, fruit expansion, and osmoregulation. Additionally, Myo-inositol derivatives influence fruit ripening, affecting flavor, texture, and nutritional value [23,31]. Its role in osmotic stress response suggests its relevance in environmental tolerance during apricot growth and maturation. In our study, the relevance of its expression is not appreciable, although it is suggested to have a greater influence on fruit development stages.

Finally, the *NAC domain-containing protein* (*NAC*) gene is part of one of the largest families of transcription factors in plants [39,40]. From the perspective of apricot, *NAC* expression could notably impact fruit ripening and quality, as well as its resistance to adverse conditions during growth, possibly represented by homogeneous levels throughout different stages in the ‘Rojo Pasion’ cultivar. For instance, *NAC* genes can regulate the expression of fruit ripening genes and ethylene synthesis, controlling ripening and improving fruit quality and shelf life [7]. Furthermore, regulating the expression of *NAC* genes can enhance abiotic stress tolerance during fruit development, improving productivity and quality under stressful environmental conditions [41]. Additionally, these genes influence cell differentiation and tissue development. In the current assay, lower *NAC* expression can affect key aspects related to fruit softening and senescence. This is reflected in the ‘Cebas Red’ cultivar, where lower NRE values compared to ‘Rojo Pasión’ result in better postharvest performance. Other evaluated genes, such as the *MADS-box protein gene* (*MADSBOX*) and *Amino-cyclopropane-1-carboxylate synthase* (*ACS*), showed very low expression values, despite these genes being related to hormonal regulation and plant development, particularly in processes such as fruit ripening. This could likely be attributed to these genes not being expressed precisely at the time of sampling, potentially occurring either before or after, as evidenced in other relevant studies [17,24].

### 3.3. Analysis of Genes Linked to Fruit Color

The *Carotenoid cleavage dioxygenase 4* (*CCD4*) gene, belonging to the *CCD* gene family, plays an essential role in carotenoid metabolism in plants, essential pigments contributing to the color of fruits and flowers and acting as precursors to important signaling molecules like abscisic acid (ABA) and volatiles influencing plant–animal interactions [42]. In apricots, *CCD4*’s function has significant implications for fruit quality. On one hand, it regulates fruit color, a key aspect of its commercial appeal and consumer perception of ripeness and quality. Additionally, its influence on volatile compound generation directly affects the aroma and thus the palatability of apricots, crucial aspects for market acceptance [43]. Lastly, *CCD4* activity can modify the composition of carotenoids, impacting the fruit’s nutritional value, as it is an important precursor for vitamin A. In this assay, ‘Rojo Pasión’ showed higher NRE values, especially during fruit growth, in contrast to ‘Cebas Red’. This could be related to the rapid fruit softening and even a higher level of aroma in ‘Rojo Pasión’ [44].

On the other hand, the *Beta-carotene-3-hydroxylase* (*CRTZ*) gene plays a crucial role in carotenoid biosynthesis by converting beta-carotene into zeaxanthin, a vital step in the metabolic pathway, generating a wide range of essential pigments responsible for the yellow, orange, and red colors in fruits, flowers, and leaves. Besides its role in coloring, carotenoids are fundamental for photosynthesis, oxidative stress protection, and the synthesis of signaling molecules like abscisic acid [45]. In relation to apricot, *CRTZ* has crucial implications for fruit development and postharvest quality. Its activity influences the intensity and hue of apricot color, a key attribute for consumer quality and ripeness perception, and also contributes to provitamin A and antioxidant content, thus enhancing the fruit’s nutritional value. Additionally, its role in protection against light stress has implications for crop adaptation to different growth conditions and for improving stress resistance [23]. In our assay, ‘Rojo Pasión’ had higher NRE than ‘Cebas Red’ in the postharvest stage, despite ‘Cebas Red’ achieving more intense orange colors. This suggests that other related genes might be controlling the color change to the more orange and reddish skin color in ‘Cebas Red’.

The *Zeta-carotene desaturase* (*ZDS*) gene plays a crucial role in carotenoid biosynthesis, acting at an early stage of the metabolic pathway leading to the production of a wide range of these pigments as well as precursors for plant hormones like abscisic acid (ABA) [46]. *ZDS* is fundamental for apricot fruit pigmentation, affecting its visual appeal and market acceptance. Furthermore, its activity influences the fruit’s nutritional content by producing carotenoids with provitamin A activity, and it may impact its ability to withstand environmental stress and maintain postharvest quality [47]. In our study, the normalized relative expression (NRE) of both cultivars increased during the postharvest period, with no differences between the cultivars.

Finally, the *UDP-glucose: flavonoid 3-O-glucosyltransferase* (*UFGT*) gene plays a crucial role in the biosynthesis of anthocyanins, water-soluble pigments responsible for red, purple, and blue colors in plants, by catalyzing the final step in this pathway by adding a glucosyl group to anthocyanidins, forming stable and soluble anthocyanins [19]. In addition to its impact on fruit, flower, and leaf coloring, this function also influences various biological functions such as UV radiation protection, attraction of pollinators and seed dispersers, and antioxidant properties [48]. *UFGT* has significant implications for several fruit quality characteristics. Its activity directly influences color intensity and hue, affecting consumer perception and market value. It also contributes to the fruit’s antioxidant properties by influencing anthocyanin accumulation, with potential health benefits for consumers [49]. In this study, the normalized relative expression (NRE) of both cultivars reached their maximum values at harvest time, with ‘Cebas Red’ showing higher levels. This likely explains the more intense orange skin color in this cultivar. Conversely, very low NRE was found for genes linked to flavonoids and anthocyanins such as *Chalcone synthase* (CHS), *Flavonoid-3’-monooxygenase* (CYP75B1), and *Anthocyanidin synthase* (ANS). 

### 3.4. Analysis of Genes Linked to the Nutraceutical Properties

The *Sucrose synthase* (SUSY) gene encodes an essential enzyme for sucrose metabolism in plants, converting sucrose into fructose and UDP-glucose, affecting physiological processes such as tissue development, stress response, and regulation of carbon flow between metabolic pathways [50]. In apricots, SUSY has significant implications for fruit quality and yield: its activity influences sugar accumulation in the fruit, affecting its sweetness and organoleptic quality, and its expression can optimize fruit size and texture to enhance marketability [31]. Additionally, SUSY’s ability to modulate stress response during fruit development can increase apricot resistance to adverse environmental conditions, potentially improving its behavior under stress. The NRE values of SUSY were clearly higher in the ‘Rojo Pasión’ cultivar, especially after harvest, which is correlated with greater fruit softening and higher sugar content compared to ‘Cebas Red’, in agreement with the observed results at phenological and RNA-seq levels [31].

The *GDP-L-galactose phosphorylase* (*VTC2_5*) gene plays a central role in ascorbic acid (vitamin C) biosynthesis in plants, being essential for both growth and development and response to environmental stress [51]. Vitamin C acts as a crucial antioxidant, protecting plant cells from oxidative damage and participating in various metabolic pathways, such as plant hormone synthesis and regulation of environmental signal responses [52]. Regarding apricots, *VTC2_5* activity has significant implications for fruit nutritional quality as it influences its vitamin C content and, therefore, its nutritional value and health benefits. Additionally, it contributes to apricot’s tolerance to environmental stress, such as drought and excess light, increasing its resistance and sustainability [53]. Furthermore, ascorbic acid plays a crucial role in extending fruit shelf life and reducing losses during storage and transportation. In our study, *GDP-L-galactose phosphorylase* displayed the highest values at the beginning of fruit growth in both cultivars, and its expression progressively decreased until the final days of postharvest. However, ‘Cebas Red’ consistently showed the highest expression, which could be linked to better postharvest performance, as observed in a 1-MCP study of the same species [28].

The *Lipoxygenase* (*LOX2*) gene encodes a fundamental enzyme called lipoxygenase, crucial in lipid metabolism in plants by oxidizing polyunsaturated fatty acids involved in physiological processes and stress responses in plants [54]. In apricots, *LOX2*’s function has significant implications for fruit quality, its resistance to stress and pathogens, and its maturation process. It affects the formation of volatile components contributing to the fruit’s aroma and flavor, so its regulation can enhance sensory quality and market appeal [55]. Additionally, its involvement in stress responses and defense against pathogens suggests that manipulating its expression could develop cultivars that are more resistant to adverse conditions and pathogen attacks [23]. No differences between cultivars were found in this study, and the NRE of the *Lipoxygenase* (*LOX2*) gene reached its minimum values at harvest and postharvest.

## 4. Materials and Methods

### 4.1. Plant Material 

The plant material used comprises eight cultivars of apricot from the genetic breeding program of CEBAS-CSIC (Murcia), in which parameters of fruit development, quality, and postharvest life were evaluated, along with the analysis of gene expression in the two most contrasting cultivars regarding these parameters. The plant materials corresponding to the eight studied cultivars (‘Cebas red’, ‘Currot’, ‘Goldrich’, ‘Orange Red’, ‘Rojo Pasión’, ‘906-12’, ‘Deseo’, and ‘Bergeron’) come from the experimental farm owned by CEBAS-CSIC, located in a single location between two municipalities in Murcia, Cieza, and Calasparra (southeast Spain, 37° N latitude, 1° W longitude, 350 m altitude) and were collected during the year 2021.

### 4.2. Experimental Design and Testing

**Figure 7:**
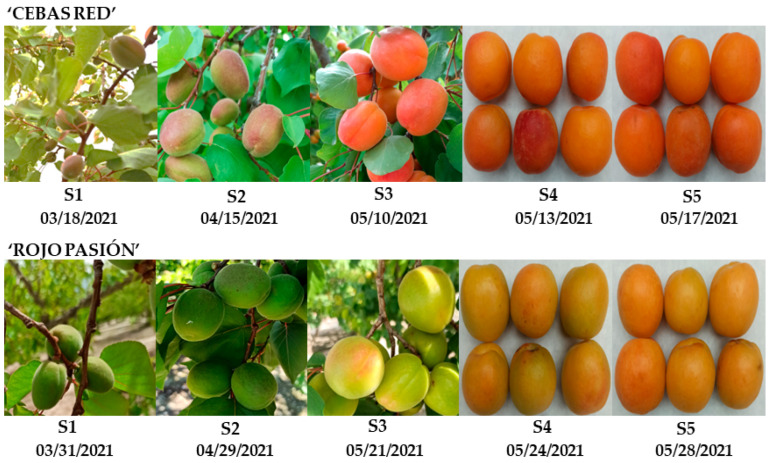
Details of the five stages (two preharvest stages S1 and S2, one harvest stage S3, and two postharvest stages S4 and S5) analyzed in this study in the apricot cultivars ‘Cebas Red’ and ‘Rojo Pasión’.

Fruit harvesting was carried out from three trees of each apricot cultivar, which were at least 10 years old and subjected to the same growing conditions. Initially, to study fruit growth and development, different branches of each cultivar were tagged for monitoring of growth and chlorophyll levels in 10 fruits across 5 developmental stages prior to harvest. Subsequently, after harvesting (commercial maturity), a trial was initiated to assess the quality and postharvest parameters throughout the fruit’s shelf life by storing the fruits under controlled conditions (20 °C) and evaluating parameters such as skin color, flesh color, chlorophyll index, and firmness in 10 fruits per day of measurement and cultivar, every 2–3 days (Figure 7). Additionally, soluble solid content and acidity were determined at harvest and at the end of the fruit’s shelf life using 3 replicates (3 samples from 3 trees), each comprising a mix of 3–4 fruits. Furthermore, respiration rate and ethylene emission were evaluated in 5 pre-selected fruits of each cultivar over 2–3-day intervals.

### 4.3. Preharvest Analysis

Fruit growth and development were monitored by measuring the equatorial diameter in mm of fruits from each cultivar previously marked using a digital Vernier caliper. For the determination of the chlorophyll index, the DA-meter equipment (Sinteléia, Bologna, Italy) was utilized. This device is a portable Vis-NIR spectrometer that correlates fruit maturity with the chlorophyll absorbance difference index known as I_AD_. Once these parameters were evaluated, the commercial harvest time was determined based on skin color and firmness parameters.

### 4.4. Postharvest Analysis

Once the fruits from each cultivar were harvested, different pomological traits, such as fruit color, were evaluated using the Minolta colorimeter (CR-300; Minolta, Ramsey, NJ, USA). Three color measurements were taken on both skin and flesh after calibration on a white porcelain reference plate. The CIELAB scale was used for color reading, determining three color coordinates (L*, a*, and b*). The Hue angle (Hº = arctangent(b*/a*)) was used for color determination. Values between 80 and 90 represent a yellow coloration, values between 70 and 80 represent an orange hue, while values below 70 represent a more reddish coloration [56]. The chlorophyll index (I_AD_) was determined using the previously described DA-meter equipment. Fruit color and chlorophyll index were evaluated on alternate days postharvest. To assess postharvest shelf life, in addition to firmness, ethylene emission was analyzed. Firmness determination was carried out using the TA.XT plus texture analyzer (Texture Technologies Corp., Kennebunkport, ME, USA), compressing an area of 5 mm^2^, resulting in the maximum force required expressed in newtons (N) for fruit deformation at a speed of 25 mm/min [31]. For ethylene production, each fruit was placed in a hermetically sealed 750 mL glass container for at least 1 h, from which 1 mL of the atmosphere was extracted using a syringe. Ethylene emission was determined in µL·kg^−1^·h^−1^ using a gas chromatograph (Konik^®^, Barcelona, Spain). The soluble solids content was determined using an Atago PAL-1 refractometer. Measurement was taken on crushed apricot pulp, and the results were expressed in ºBrix [31]. For acidity evaluation, 2 g of crushed sample were weighed and diluted in 30 mL of distilled water. Using an automatic titration system (model 785 DMP Tinitro Metrohm Ltd., Herisau, Switzerland), acidity was determined by neutralization with 0.1 N NaOH until a pH of 8.1 was reached. Results were expressed in grams of malic acid/100 mL. The soluble solids content and acidity were evaluated at harvest and on the final postharvest day

### 4.5. RNA Isolation and Purification 

Measurements were taken at each stage during the stages of fruit growth and development as well as throughout the postharvest period, and fruit samples were frozen at −80 °C. Finally, ‘Cebas Red’ and ‘Rojo pasión’ were selected as the most contrasting cultivars in terms of firmness and ethylene production during postharvest, considering 5 different states, for RNA extraction (Figure 7): 2 preharvest, 1 harvest, and 2 postharvest states. For RNA extraction, three replicates were considered from a group of fruits for each of the analyzed development and ripening states. The samples were preserved in liquid nitrogen for grinding before the extraction procedure, thus avoiding sudden temperature changes to prevent damage to cellular metabolism. Fruit grinding was performed using a grinder (KIA^®^ A11 basic Analytical Mill, Barcelona, Spain), resulting in a very fine powder. Samples in this state were stored in Eppendorf tubes at −80 °C. RNA extraction from the powdered samples is based on an adaptation of the protocol [57]. In this protocol, 2% CTAB buffer, chloroform/isoamyl alcohol, 24:1, and 10 M LiCl are used. CTAB is a detergent that precipitates proteins and polysaccharides; β-mercaptoethanol is an RNase inhibitor; chloroform/isoamyl alcohol 24:1 denatures proteins and separates them from nucleic acids, and finally, LiCl induces RNA precipitation. Following RNA extraction, agarose gel electrophoresis was performed to assess the integrity and purity of the RNA, aiming to evaluate the quality of the extractions. RNA was concentrated and purified using the RNeasy^®^ PowerClean^®^ Pro Cleanup Kit from Qiagen (Hilden, Germany). The purification protocol was based on the use of a series of washing solutions to leave the RNA free from any contaminants. Once purified RNA was obtained, its concentration and purity were evaluated using a UV-visible microvolume spectrophotometer called Thermo Scientific™ NanoDrop™ (Madrid, Spain). This equipment provided A260/A280 nm and A260/A230 nm absorbance ratios, with optimal values around 2, indicating adequate RNA purity.

### 4.6. Gene Expression Analysis Using RT-qPCR

First, cDNA synthesis was performed using the SimpliAmp thermocycler (Applied Biosystems^®^, Norwalk, CT, USA). Starting with 500 ng of RNA, 20 μL of cDNA at a concentration of 50 ng/μL was obtained. The volume of RNA needed to reach 500 ng was added, and water was added to reach a volume of 11 μL. Additionally, 1 μL of dNTPs (10 mM) and 1 μL of oligo(dT) (50 mM) were added, resulting in a final volume of 13 μL. The mixture was incubated in the thermocycler at 65 °C for 5 min, followed by 1 min on ice. After this step, the following reagents were added: 4 μL of 5x SSII buffer, 1 μL of 0.1 M DTT, 1 μL of RNaseOUT, and 1 μL of SuperScript III Reverse Transcriptase (Thermo Fisher Scientific, Norwalk, CT, USA), reaching a final volume of 20 μL. Reverse transcription was performed in the thermocycler. For real-time qPCR, a 10 μL mixture was prepared, including 5 μL of Power SYBR^®^ Green PCR Master Mix (Applied Biosystems, Norwalk, CT, USA), 0.5 μL of each primer (5 μM), and 2 μL of cDNA. The amplification protocol for qPCR using the StepOnePlus Real-Time PCR System (Applied Biosystems, Norwalk, CT, USA) was as follows: 10 min at 95 °C for initial cDNA denaturation followed by 40 cycles of 15 s at 95 °C, 1 min at 60 °C, and for melting curves, 15 s at 95 °C, 1 min at 60 °C, and a final temperature increase in small intervals of 0.3 °C up to 95 °C for 15 s [23]. The assay was performed in 96-well plates using three biological replicates (3 samples from 3 trees) and two technical replicates. Before gene expression evaluation, standard curves were generated to determine the optimal cDNA concentration to use, as well as the efficiency of each primer. Dilutions were made to 1.25 ng/μL, 2.5 ng/μL, 5 ng/μL, and 10 ng/μL, starting from 20 μL of cDNA at 50 ng/μL. Finally, a concentration of 2.5 ng/μL of cDNA was considered suitable for monitoring the expression of each gene. During the qPCR process, product amplification is quantified by fluorescence, where a lower Ct value indicates higher gene expression. Relative Normalized Quantification (NRQ) was obtained for all evaluated genes using the modified Pfaffl method [58], which determines gene expression levels relative to an internal (housekeeping genes) control or reference gene. Nineteen genes related to fruit growth and ripening processes were evaluated with respect to four internal controls. The evaluated genes were: *polygalacturonase* (*PG*), *pectinmethylesterase* (*PME*), *auxin-sensitive protein* (*IAA*), *ferredoxin* (*PET*), *beta-carotene-3-hydroxylase* (*CRTZ)*, *GDP-L-galactose phosphorylase* (*VTC2_5*), *3-hydroxyisobutyryl-CoA hydrolase* (*HIBCH*), *sucrose synthase* (*SUSY*), *aminocyclopropane-1-carboxylate synthase* (*ACS*), *flavonoid-3’-monooxygenase* (*CYP75B1*), *myo-inositol-1-phosphate synthase* (*INO1*), *carotenoid cleavage dioxygenase* (*CCD4*), *ζ-carotene desaturase* (*ZDS*), *chalcone synthase* (*CHS*), *lipoxygenase* (*LOX2*), *NAC gene* (*NAC*), *anthocyanidin synthase* (*ANS*), *UDP-glucose: flavonoid 3-O-glucosyltransferase* (*UFGT*), and *MADS-box gene* (*MADSBOX*) (Supplementary Table S1). The four housekeeping genes used were *RNA polymerase II* (*RPII*), *actin*, *TEFII,* and *Ubiquitin 10* (*UBQ10*). These candidate genes were identified as key genes in a recent RNA-Seq study conducted on apricot fruits [31].

### 4.7. Data Analysis

To identify significant differences between the various cultivars and ripening stages, ANOVA analysis and Tukey’s test (*p*-value ≤ 0.05) were employed. These statistical analyses were performed using INFOSTAT v18 software (Universidad Nacional de Córdoba, Córdoba, Argentina).

## 5. Conclusions

Gene expression during fruit development, ripening, and postharvest was monitored in apricot fruits at various stages (two preharvest stages S1 and S2, one harvest stage S3, and two postharvest stages S4 and S5) using advanced RT-qPCR techniques. This approach provided a deeper understanding of the biochemical and physiological changes occurring during these critical periods. Furthermore, the study successfully established correlations between gene expression and phenotypic traits. This correlation is crucial for developing effective strategies for enhancing fruit quality. Genes associated with fruit firmness (*PG* and *PME*) and color (*CCD4*, *CRTZ*, *ZDS*, and *UFGT*), which are essential for improving the shelf life and fruit quality, were identified and characterized. Additionally, we identified differential expression of genes related to aromatic and nutraceutical aspects such as *VTC2_5*, *SUSY*, and *LOX2*. These findings open new avenues for future research, particularly in enhancing the nutritional and sensory quality of apricots. In this context, this study represents a significant step towards understanding and improving apricots, combining traditional phenological methodologies with advanced molecular tools to address the challenges of modern agriculture and market demands. 

## Figures and Tables

**Table 1:** Summary of descriptive statistics for quality traits of fruits evaluated at harvest across the eight assayed apricot cultivars.

Apricot Cultivar	Trait	Mean ± SD	Apricot Cultivar	Trait	Mean ± SD
**‘Cebas Red’**	Ripening date	130	**‘Orange Red’**	Ripening date	153
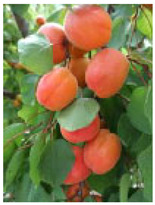	Fruit weight	63.03 ± 6.77	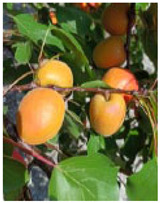	Fruit weight	61.04 ± 6.39
	I_AD_	0.31 ± 0.13		I_AD_	0.95 ± 0.23
	Skin color	74.02 ± 3.39		Skin color	77.05 ± 2.97
	Blush color	52.80 ± 8.94		Blush color	49.87 ± 15.97
	% Blush color	24.50 ± 13.22		% Blush color	25.50 ± 14.42
	Flesh color	73.93 ± 1.03		Flesh color	73.93 ± 2.47
	Firmness	76.83 ± 15.59		Firmness	53.41 ± 7.06
	Ethylene	0.08 ± 0.01		Ethylene	0.33 ± 0.11
	SSC	7.97 ± 0.06		SSC	10.40 ± 0.46
	Acidity	1.10 ± 0.20		Acidity	1.07 ± 0.06
**‘Currot’**	Ripening date	132	**‘Goldrich’**	Ripening date	158
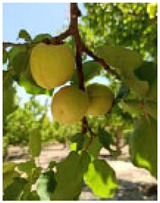	Fruit weight	36.83 ± 5.52	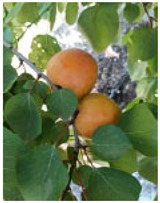	Fruit weight	126.63 ± 14.37
	I_AD_	0.73 ± 0.18		I_AD_	0.72 ± 0.22
	Skin color	105.40 ± 2.07		Skin color	77.87 ± 1.72
	Blush color	82.93 ± 8.91		Blush color	69.42 ± 4.53
	% Blush color	10.00 ± 5.27		% Blush color	13.00 ± 10.85
	Flesh color	98.00 ± 3.37		Flesh color	72.55 ± 1.19
	Firmness	108.90 ± 20.57		Firmness	73.42 ± 14.41
	Ethylene	0.11 ± 0.08		Ethylene	0.35 ± 0.33
	SSC	11.07 ± 0.73		SSC	10.33 ± 0.06
	Acidity	1.76 ± 0.15		Acidity	2.65 ± 0.34
**‘Rojo Pasión’**	Ripening date	141	**‘Deseo’**	Ripening date	167
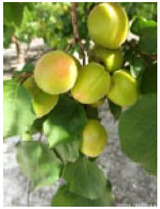	Fruit weight	71.39 ± 9.59	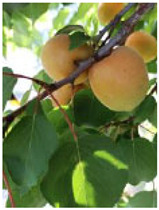	Fruit weight	65.29 ± 6.58
	I_AD_	1.14 ± 0.21		I_AD_	1.16 ± 0.18
	Skin color	84.01 ± 3.31		Skin color	75.05 ± 2.01
	Blush color	77.12 ± 7.66		Blush color	52.80 ± 7.32
	% Blush color	7.50 ± 4.25		% Blush color	17.50 ± 10.34
	Flesh color	75.94 ± 1.54		Flesh color	73.57 ± 1.44
	Firmness	24.65 ± 10.79		Firmness	56.61 ± 8.24
	Ethylene	32.57 ± 15.69		Ethylene	0.27 ± 0.19
	SSC	9.60 ± 0.75		SSC	12.90 ± 0.44
	Acidity	1.13 ± 0.08		Acidity	1.40 ± 0.02
**‘906-12’**	Ripening date	146	**‘Bergeron’**	Ripening date	174
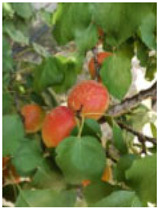	Fruit weight	84.38 ± 5.54	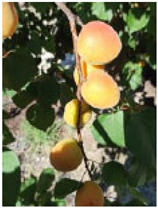	Fruit weight	55.66 ± 4.66
	I_AD_	0.86 ± 0.08		I_AD_	0.73 ± 0.16
	Skin color	84.45 ± 2.33		Skin color	84.59 ± 3.23
	Blush color	49.22 ± 10.32		Blush color	71.05 ± 8.68
	% Blush color	27.50 ± 8.90		% Blush color	14.50 ± 7.25
	Flesh color	80.58 ± 1.87		Flesh color	75.89 ± 2.37
	Firmness	67.94 ± 12.19		Firmness	42.99 ± 8.26
	Ethylene	0.36 ± 0.30		Ethylene	0.26 ± 0.13
	SSC	11.03 ± 0.21		SSC	10.13 ± 0.47
	Acidity	1.45 ± 0.08		Acidity	1.87 ± 0.14

## Data Availability

Data are contained within the article and Supplementary Materials.

## Supplementary Materials

The following supporting information can be downloaded at: https://www.mdpi.com/article/10.3390/ijms25169081/s1.
