# Supplementary material for: Monitoring Fruit Growth and Development in Apricot (Prunus armeniaca L.) through Gene Expression Analysis
Source: Int J Mol Sci. 2024 Aug 21;25(16):9081. doi: 10.3390/ijms25169081 (PMC11354700; doi:10.3390/ijms25169081)
Supplement: Supplementary file 1 [file ijms-25-09081-s001.zip › ijms-3104445-supplementary.pdf]

Supplementary Table S1. Descriptive table of the studied candidate genes. Gene id, function and primer sequence.

Gene	Gene id. (*P. Persica*)	Gene id. (*P. Armeniaca*)	Function	Forward	Reverse
Auxin-sensitive protein (IAA)	Prupe_8G215400	ChrG8: 18244819 - 18250027	Involved in hormonal signaling, particularly in auxin-mediated growth and development.	TCAGAGAAGGCGTACCAAGAA	CTTGCTCCAAGGCTACAGGA
Ferredoxin (PET)	Prupe_1G573600	ChrG1: 44649984 - 44650748	Participates in photosynthetic processes and electron transfer, and energy metabolism.	ACCAAACATGGCAGCTCTCT	CGCAGCGTCAAGAATGTAAA
Myo-inositol-1-phosphate synthase (INO1)	Prupe_6G180500	ChrG6: 13170206 - 13173800	Participates in the synthesis of myo-inositol, important for carbon storage and osmoregulation.	CCAGATGACGTTGTGTTTGG	GCACCTTGGTTAGCAGCAAT
MADS-box protein gene (MADSBOX)	Prupe_1G549600	ChrG1: 42319590 - 42322858	Involved in the regulation of floral differentiation and fruit development.	GATGGGGAGAGGAAAGATTGA	CTTCAGCAGTCCATTTCTTCG
NAC domain-containing protein (NAC)	Prupe_4G186800	ChrG4: 12814165 - 12816027	Regulates the expression of genes involved in fruit development and maturation.	AGAACTCAGCGGGTTGATAACT	TGCACCCCTACTCGATTTCT
Polygalacturonase (PG)	Prupe_4G261900	ChrG4: 23028346 - 23030086	Participates in the degradation of pectin during fruit maturation, affecting fruit softening.	ATGCAAGGTGTCAGGGTTTC	CATTTTGTACACCGGCCTCT
Pectin methylesterase (PME)	Prupe_7G192800	ChrG7: 17725152 - 17726476	Modifies pectin in the fruit cell walls, influencing the firmness and texture of the fruit.	CACGTTGTGTGACGATAGGG	ACAAAGGCAAACCCACTGTC
Aminocyclopropane-1-carboxylate synthase (ACS)	Prupe_7G213900	ChrG7: 18984148 - 18985965	Essential in the biosynthesis of ethylene, a hormone that regulates fruit maturation and senescence.	CTTCCAACTGGGGATCAAAA	AAAGCTCATTGGCTGCAGTT
Anthocyanidin synthase (ANS)	Prupe_5G086700	ChrG5: 8132544 - 8134462	Participates in the synthesis of anthocyanins, pigments that contribute to the fruit's color.	GCTCAATGGGAAGATCGAAA	AGGAGTTGAAGAAGGCAGCA
UDP-glucose: flavonoid 3-O-glucosyltransferase (UFGT)	Prupe_6G190100	ChrG6: 16834730 - 16837246	Involved in the glucosylation of anthocyanins, affecting the stability and color of the fruit.	TCAGGCCTAACAATCCACAA	GAATGTTTGAAATGGCTTGA
Beta-carotene-3-hydroxylase (CRTZ)	Prupe_2G300800	ChrG2: 27654116 - 27655815	Intervenes in the synthesis of carotenoids, compounds that contribute to color.	TGAGGAGGGCTATAGCTGGA	ATGGAGTTTTGGGCAAGATG

Carotenoid cleavage dioxygenase 4 (CCD4)	Prupe_1G255500	ChrG1: 24120819 - 24123431	Implicated in the degradation of carotenoids, affecting the color and aroma of the fruit.	TCGGTTGAGAAAGTTAGGATTGATC	CGGATTGAACACAGCAAAGTCTA
Zeta-carotene desaturase (ZDS)	Prupe_6G340000	ChrG6: 24962662 - 24965799	Participates in the synthesis of carotenoids, essential for the color and nutritional content of the fruit.	TGGGATGATGGGAATAAAGC	GGACAAGGTTCACTCCTCCA
Chalcone synthase (CHS)	Prupe_I005800	ChrG1: 371685 - 373349	Implicated in the synthesis of flavonoids, affecting the color, taste, and disease resistance.	TGGGTGTGCAATCCAGAATA	CCGTGAAGTTGGGCTTACAT
Flavonoid-3′-monooxygenase (CYP75B1)	Prupe_5G203600	ChrG5: 15462033 - 15463374	Intervenes in the biosynthesis of flavonoids, affecting the color and antioxidant properties of the fruit.	GTGCATGAGAGGCCCATACT	CGCCTTCTTGTACCGTCTTC
3-hydroxyisobutyryl-CoA hydrolase (HIBCH)	Prupe_2G207600	ChrG2: 22380189 - 22381851	Implicated in the metabolism of branched-chain amino acids, affecting the aroma.	TGCCCTTGAGAAGGAGGTTA	CTGTACTCGCGGACAAGACA
GDP-L-galactose phosphorylase (VTC2_5)	Prupe_4G220800	ChrG4: 16618604 - 16621796	Participates in the synthesis of ascorbic acid (vitamin C), important for the nutritional value.	CAAGCTCTTGGGGAAGTGAG	CAAGCAATGGCCTCAAAAAT
Sucrose synthase (SUSY)	Prupe_7G192300	ChrG7: 17698863 - 17702345	Participates in sucrose metabolism, influencing the sweetness and energy development of the fruit.	AAGGGTATTGTCCGCAGATG	TCAATGAGGCCACAATGTTC
Lipoxygenase (LOX2)	Prupe_4G047800	ChrG4: 2655575 - 2658319	Related to the fatty acid pathway and synthesis of volatile compounds, influencing the aroma.	CTGGTACAACGAGTCAATCAATTTAGG	AATGGATGTAAGATCATCTGGAGTAGAG
RPPII	--	--	Housekeeping gene	TCCTGAGGCATACACATAC	CATTGAGCACCAACTGAG
Actin	--	--	Housekeeping gene	CTGGATTCTGGTGATGGT	GCAGTTGTTGTGAAGGAG
TEFFII	--	--	Housekeeping gene	CGTGACTTCATCAAGAACATG	CCGATCTGTACGTCCTG
UBQ10	--	--	Housekeeping gene	GCCGCACTCTTTCTGACTACAAC	ACCTCCAGAGTGATGGTCTTGC

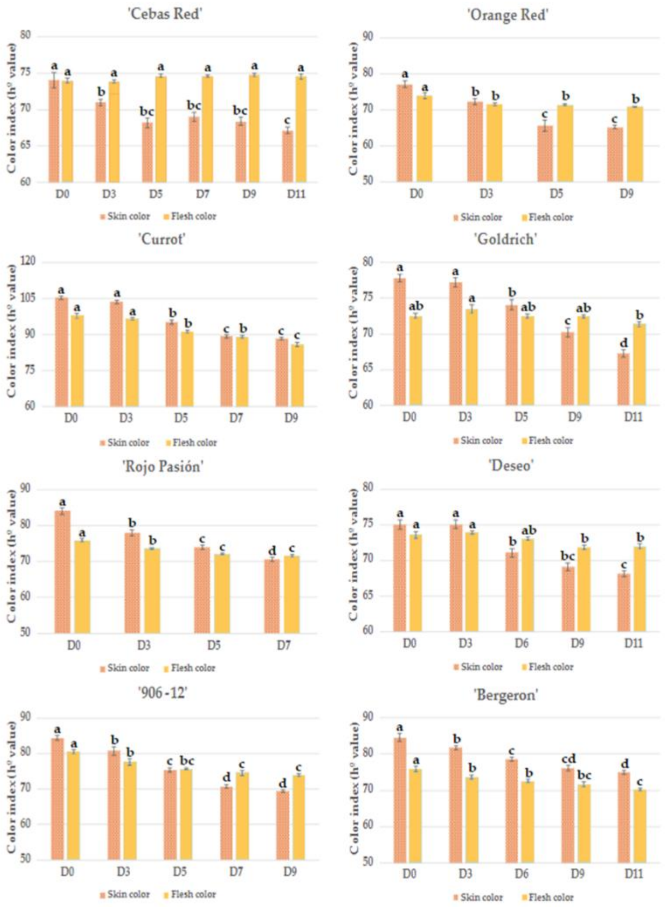

Supplementary Figure S1. Skin color (red bars) and flesh color (orange bars) during the post-harvest period of the trial across 8 apricot cultivars. Different letters indicate significant differences between the days for each genotype according to the Tukey test (p-value < 0.05).

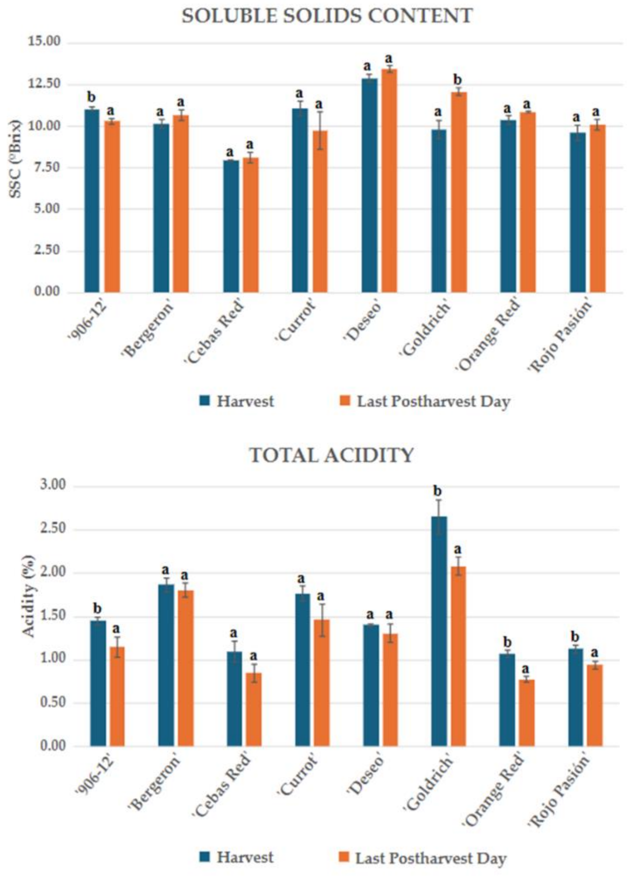

Supplementary Figure S2. Soluble solids content (up) and total acidity (down) at harvest and at the end of the post-harvest period of the trial across 8 apricot cultivars. Different letters indicate significant differences between the days for each genotype according to the Tukey test (p-value < 0.05).
